# Promiscuous Effects of Some Phenolic Natural Products on Inflammation at Least in Part Arise from Their Ability to Modulate the Expression of Global Regulators, Namely microRNAs

**DOI:** 10.3390/molecules21091263

**Published:** 2016-09-21

**Authors:** Esmerina Tili, Jean-Jacques Michaille

**Affiliations:** 1 Department of Anesthesiology, Wexner Medical Center, The Ohio State University, Columbus, OH 43210, USA; 2 Department of Cancer Biology and Genetics (CBG), Wexner Medical Center, The Ohio State University Wexner Medical Center and Comprehensive Cancer Center, Columbus, OH 43210, USA; Jean-Jacques.Michaille@u-bourgogne.fr; 3 BioPerox-IL, UB-INSERM IFR #100, Université de Bourgogne-Franche Comté, Faculté Gabriel, 6 Bd. Gabriel, 21000 Dijon, France

**Keywords:** auto-immune disorders, cancer, cardiovascular pathologies, inflammation, microRNAs, *miR-155*, resveratrol

## Abstract

Recent years have seen the exploration of a puzzling number of compounds found in human diet that could be of interest for prevention or treatment of various pathologies. Although many of these natural products (NPs) have long been used as remedies, their molecular effects still remain elusive. With the advent of biotechnology revolution, NP studies turned from chemistry and biochemistry toward global analysis of gene expression. Hope is to use genetics to identify groups of patient for whom certain NPs or their derivatives may offer new preventive or therapeutic treatments. Recently, microRNAs have gained the statute of global regulators controlling cell homeostasis by regulating gene expression through genetic and epigenetic regulatory loops. Realization that certain plant polyphenols can modify microRNA expression and thus impact gene expression globally, initiated new, mainly in vitro studies, in particular to determine phytochemicals effects on inflammatory response, whose exacerbation has been linked to several disorders including cancer, auto-immune, metabolic, cardiovascular and neuro-inflammatory diseases. However, very few mechanistic insights have been provided, given the complexity of genetic regulatory networks implicated. In this review, we will concentrate on data showing the potential interest of some plant polyphenols in manipulating the expression of pro- and anti-inflammatory microRNAs in pathological conditions.

## 1. Introduction

Inflammation corresponds to a set of multiple, non-specific reactions developed by the body to defend against pathogens, allergens, toxic or irritant molecules, or to deal with damaged cells and participate to the repair of tissue injuries [1]. The inflammatory response is regulated by a vast set of mediators, including the production of cytokines and chemokines allowing the recruitment and/or activation of different types of leukocytes, the production of nitrogen reactive species, or the activation of specific enzymes implicated in the production of a number of mediators such as prostaglandins, leukotrienes, matrix metalloproteases or other factors. Inflammation is normally contained, its termination being controlled by some of the mediator molecules produced. In certain pathological conditions, however, inflammation can become unrestrained and spread to other tissues via the circulatory and/or the lymphatic system, that can lead to a systemic inflammatory syndrome such as sepsis [1]. In other cases, inflammation can become chronic, and this chronicity has been linked with a wide range of pathologies, including, but not limited to, rheumatoid arthritis, inflammatory bowel disease, chronic asthma, multiple sclerosis and other auto-immune diseases, metabolic syndrome and cancer [2].

**Figure 1:**
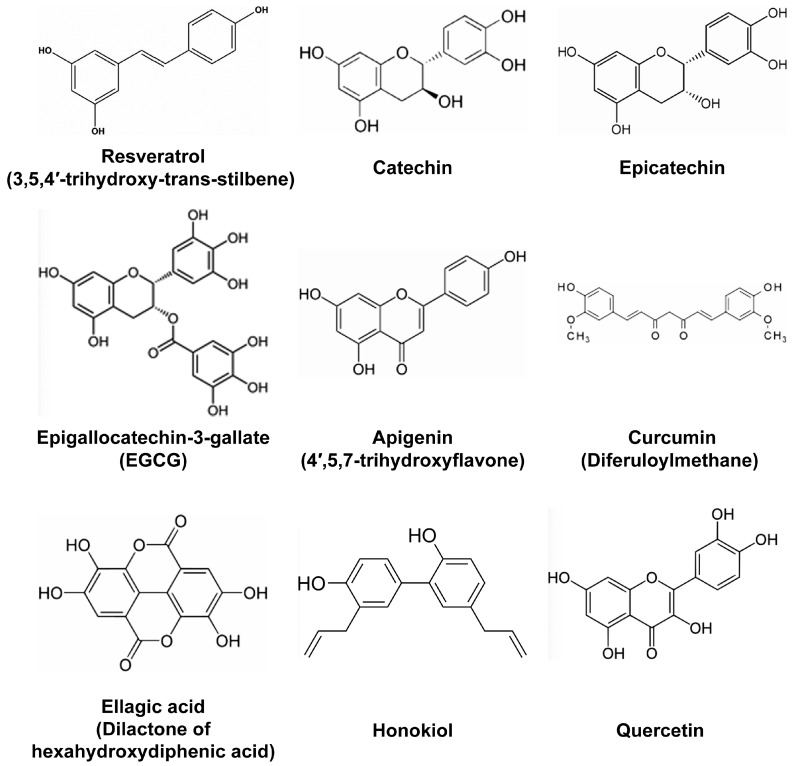
Structure of the main plant polyphenols discussed in this review.

It is therefore of tremendous interest to identify molecules presenting anti-inflammatory properties. Many plants have been known for a long time to contain compounds with anti-inflammatory properties, and their study has recently gained more interest with the demonstration of their potential multiple beneficial effects to the body in helping it defend against pathogens and diseases as well as in preventing or delaying a number of inflammatory-related pathologies. In this review, we will discuss the effects of some polyphenols that are secondary metabolites of plants, such as those presented in Figure 1, many of which are commonly present in human diet.

Among the many active natural compounds, resveratrol (3,4′,5-trihydroxystilbene), a polyphenol mostly found in grape and other berries, green tea polyphenols such as epigallocatechin-3-gallate (EGCG), curcuminoids (found in the Indian spice known as turmeric), honokiol (a polyphenol found in the bark, seed cones and leaves of Magnolia tree), flavonoids such as proanthocyanidins found in different berries and cocoa that are derived from flavon-3-ols such as catechin or epicatechin, apigenin (4′,5,7-trihydroxyflavone), found in parsley, chamomile or chilly, quercetin, propolis (found in bee resin and rich in flavonoids), and other phenolic compounds, but also NPs from different chemical classes such a terpenoids, have been shown to exert anti-inflammatory effects in different pathological conditions (Figure 1) [3,4,5,6,7,8,9,10,11,12].

These effects were primarily measured by assessing “typical” pro-inflammatory markers such as Tumor necrosis factor (TNF), Interleukin (IL)-1β, IL-6, as well as the activation of signal transduction pathways such as Mitogen activated protein (MAP) kinases, nuclear factor kappa B (NF-κB), transforming growth factor (TGF)-β, or AKT. Simultaneously, most of the above phenolic NPs show strong anti-oxidant activity that has been attributed to their effects in modulating the expression of different radical scavenging enzymes involved in oxidative stress response, such as Superoxide dismutase or Glutathione peroxidase [13]. Additional beneficial effects of NPs found in our diet, are related with their effects on gut microbial composition [14], specifically affecting the gut inflammation. Finally, the flavonoid apigenin was reported to inhibit hepatitis C virus replication [15]. Although extracts from different plants were used, the effects of these phytochemicals under different chronic inflammatory conditions were quite similar. This either reflects the fact that most of the plant extracts used contain the same polyphenols or polyphenols with similar structure as their active ingredients, that these polyphenols have multiple common non-specific effects arising from their related molecular structures, or alternatively, that these phytochemicals interfere with the same signal transduction pathway(s), and modulate common regulators of inflammation. It is probable that polyphenols might bind to and impair the function of some of the microRNAs involved in different inflammation-related conditions. However, distinctive differences between plant phenolic compounds may possibly exist with regard to their biological function that would lead to the preferential use of a given compound for a particular pathology. It is therefore essential that the research extends toward elucidating the specific effects of plant polyphenols, or identify the main active compounds of plant extracts that have anti-inflammatory effects, as it is possible that different active compounds of the same extract might have opposing effects within the broad magnitude of factors involved in inflammation. Many presently available resources/technologies should allow identifying the specific effects of some of the candidate plant polyphenols, on a given transcription factor, enzyme, RNA-binding protein, microRNA, or metabolite. Combination of global analyses will give us a better understanding of specific effects of different phenolic NPs, crucial for personalized and cell-specific directed future therapies. We first will review the most recent publications on the subject of certain plant polyphenols and inflammation. Next, we will discuss the effects of such phytochemicals in inflammation from the microRNAs point of view. Finally, we will propose our view on this subject, and point to future directions. For additional reading on microRNAs and inflammation, please consult our previous review [16].

## 2. Phytochemicals and Inflammatory Factors

- Resveratrol (Figure 1) is probably the most studied polyphenol with anti-inflammatory properties. It has been proposed that in addition to being ingested with food derived from grapes and other berries, resveratrol could possibly be used as a cream for the treatment of epithelium lesions such as dermatitis. Indeed, in a murine model of atopic dermatitis-like lesions induced by the application of 2,4-dinitrofluorobenzene, systemic administration of resveratrol ameliorated the outcome by reducing the expression levels of epithelium derived cytokines, such as IL-25, IL-33, and thymic stromal lymphopoietin [17]. Reduced levels of these factors following resveratrol treatment was associated with the reduction of apoptotic epithelial cells as measured by the levels of caspase-3 and the increased thickness of the epithelium [17]. In another study, the polyphenols-rich fraction of grapes reduced the metabolic consequences of high-fat diet by decreasing adiposity and insulin resistance in addition to reducing inflammatory markers [14]. Interestingly, the same extract also impacted the gut microbiota in high fat-fed mice, as mice fed on these extracts showed alterations in gut microbiota groups. Another study found that resveratrol improved renal injury caused by micro-inflammation in a hypertensive renal damage rat model [18]. In this model, resveratrol exerted anti-fibrotic functions by reducing the levels of Plasminogen activator inhibitor (PAI)-1, TGF-β, and Fibronectin [18]. Of note, our group previously reported that TGF-β pathway is the primary target of resveratrol in human colon cancer cells [19]. We came to this conclusion, based on the fact that resveratrol changed the expression of different microRNAs targeting effectors of TGF-β signaling, including TGF-β itself [19]. Resveratrol anti-fibrotic effects in the hypertensive renal damage rat model were complemented with anti-inflammatory effects, as the expression of NF-κB, IL-6, Intercellular adhesion molecule (ICAM)-1 and Monocyte chemoattractant protein-1 (MCP) was significantly reduced [18]. Based on its anti-inflammatory properties, a combinatorial use of resveratrol with non-steroidal anti-inflammatory drug (NSAID) ibuprofen was tested for the ability to diminish innate immune response on lipopolysaccharide (LPS)-challenged RAW-264.7 mouse macrophages [20]. In parallel, different resveratrol-NSAID derivatives were tested in the same conditions, and one of these derivatives was found to present stronger anti-inflammatory properties than resveratrol, ibuprofen or their mixture. As mentioned earlier, resveratrol has been known to also reduce neuro-inflammation and might be of interest for slowing the progression of neuro-inflammatory pathologies. For example, resveratrol was tested for memory enhancing and its effects on cognitive disorders such as Alzheimer’s disease in a mouse model of this pathology [21]. In Alzheimer’s disease, Beta amyloid (Aβ) peptides are implicated in cognitive impairment, neuro-inflammation, and neuronal apoptosis [21]. Therefore, the effects of resveratrol were tested in mice microfused with Aβ peptides to induce learning/memory impairment, neuro-inflammation and apoptosis. Resveratrol was found to reverse Aβ1-42-induced expression of Phosphodiesterase 4A, 4B and 4D, and reverse Aβ1-42-induced decrease in the phosphorylation level of cAMP response-element binding protein (CREB), Brain derived neurotrophic factor (BDNF) and Anti-apoptotic factor B-cell CLL/lymphoma (Bcl)-2 [21].
- Curcuminoids, with curcumin (Figure 1) being the most studied, are known for their anti-inflammatory and free radical-scavenging activities. Among critical discoveries was the study that found curcumin supplementation to significantly reduce circulating levels of TNF in randomized clinical trials using meta-analysis [22]. In rats, curcumin was found to alleviate renal dysfunction and suppress inflammation in daunorubicin-induced nephrotoxicity by shifting macrophage polarization toward the M2 phenotype [23]. This shift of macrophage polarization to M2 phenotype in the presence of curcumin was due to the reduction of Extracellular signal–regulated kinase (ERK)1/2 and NF-κB p65 expression, concomitant to the upregulation of IL-10 expression [23]. In another study, curcumin significantly reduced oxidative stress and inflammation in a rat model of gastric antral ulceration [24]. In curcumin-fed rats, the inhibition of lipid peroxidation and naproxen-induced gastric ulcer was attributed to the effects of curcumin in increasing the activities of scavenging enzymes, such as Superoxide dismutase, Catalase, and Glutathione peroxidase [24]. The effects of polyphenols found in French *Curcuma longa*, were tested, in 3T3-L1 adipocytes under oxidative stress [25]. These polyphenols improved insulin-mediated lipid accumulation, and increased *Peroxisome proliferator-activated receptor-gamma* (*PPARγ*) gene expression and adiponectin secretion. The same polyphenols also impaired oxidative stress-induced production of IL-6, TNF, MCP-1, and NF-κB [25]. Expectedly, these polyphenols also reduced intracellular levels of ROS and modulated the expression of genes encoding Superoxide dismutase (SOD) and Catalase antioxidant enzymes [25]. Additionally, curcumin, was tested, for its fungicidal effects on *Sporothrix schenckii* fungus [26]. This study found that sublethal curcumin treatments of *S. schenckii* activate protein kinase C (PKC) and increase the expression of Chitin synthase 1 and 3, resulting in increased chitin content on the *S. schcenckii* conidial cell wall [26]. As a result, mice infected with curcumin-treated conidia showed reduced fungal burden. Thus, like resveratrol, curcumin presents with a wide range of anti-inflammatory effects, suggesting that both compounds might affect the expression of microRNAs involved in immune response and inflammation.
- Tannin-rich fruits tested for their anti-inflammatory effects, in a preclinical model of colitis, showed protective effects against ulcerative colitis. Beverages prepared from mango and pomegranate (*Punica granatum*) rich in gallotannins and ellagitannins, respectively, were tested in a rat sulfate-induced colitis model [27]. While both beverages reduced intestinal inflammation and the levels of pro-inflammatory cytokines in mucosa and serum, they also showed specific effects. Thus, mango beverage impaired the strong activation of Insulin-like growth factor (IGF)-1/AKT/rapamycin (mTOR) pathway, by reducing the expression of *IGF1* and *Insulin receptor* (*INSR*) genes, while pomegranate reduced the levels of Ribosomal protein S6 kinase beta-1 (p70S6K), Ribosomal protein S6 (RPS6), as well as those of transcripts encoding Ribosomal protein S6 kinase A2 (RPS6KA2), Mitogen-activated protein kinase kinase 2 (MAP2K2), and MAPK1 [27]. Finally, polyphenolic extracts prepared from these two beverages inhibited IGF-1R/AKT/mTOR pathway (mango) or ERK1/2-mTOR pathway (pomegranate), respectively [27]. In addition, in silico analysis showed the presence of a high binding-dock of gallic acid to the catalytic domain of IGF-1R, while ellagic acid could dock effectively into Epidermal growth factor receptor (EGFR) and IGF-1R, opening the possibility to use gallic acid to specifically impair EGFR pathway [27]. These data point to specific effects of these two-tannin rich fruits in colitis, and suggest that a combination of both extracts potentially could have complementary protective effects against ulcerative colitis. This study supports the hypothesis that active compounds found in our diet, although highly similar, might have specific effects on cellular signaling and pathways they modulate in the cell. In addition, their activity depends on the “healthy” state of the cell, so that we cannot expect phenolic NPs to have identical effects in normal versus pathological conditions on a given cell.
- EGCG, the most abundant polyphenol of green tea, has widely been studied for its anti-oxidant, anti-cancer and anti-inflammatory effects. The anti-inflammatory effects of EGCG are of particular interest for treatment of inflammatory bowel disease (IBD) [28]. In inflamed colon, EGCG reduces myeloperoxidase activity and production of pro-inflammatory factors, and provides a general protection of gut mucosa [29]. From a metabolic point of view, green tea phytochemicals reduce body mass index and waist circumference and improve lipid metabolism [30]. In another study, EGCG reduced inflammation and pain caused by intervertebral disc degeneration [31]. Interestingly, in addition to reducing inflammation, EGCG enhanced the survival of primary human disc cells under oxidative stress. Furthermore, EGCG enhanced the survival of the same cells in lethal oxidative stress conditions through activation of pro-survival PI3K/AKT pathway. It will be important to determine parameters that allow the tested compounds to be either pro-apoptotic (thus being beneficial for cancer treatment) or anti-apoptotic (thus protecting cells from death induced by ischemic or oxidative stress).
- Anti-inflammatory effects of polyphenols found in cocoa extracts were tested on mouse RAW-264.7 macrophages challenged with LPS to induce innate immune response [32]. The reaction of RAW-264.7 macrophages to LPS was significantly attenuated by cocoa extracts polyphenols, as measured by reduced levels of pro-inflammatory enzymes such as 5-Lipoxigenase (5-LOX), prostaglandin E2 (PGE2), reactive oxygen species (ROS), nitric oxide (NO), and TNF [32]. Analogous experiments with similar outcomes were conducted with phenolic and terpene extracts from the leaves of different species of *Oregano* [33]. Additionally, cocoa supplements, were reported to improve endothelial cell function [34], as did *Chilean propolis* [35], or resveratrol on human colon-derived CCD-18Co myofibroblast cells [36].
- The serum of mice pre-treated with banana peel polyphenols presented with reduced levels of pro-inflammatory cytokines such as TNF, IL-6, IL-1β, and IL-12 following treatment with carbon tetrachloride, a molecule used to induce hepatic injury [37]. Other inflammatory-related factors such as COX-2, and NO were also reduced. Banana peel polyphenols likewise reduced serum levels of Aspartate aminotransferase, Alanine aminotransferase and Lactate dehydrogenase enzymes. Overall, the capability of banana peel polyphenols to reduce the extent of carbon tetrachloride-induced liver injury and to protect liver cells, were attributed to their anti-inflammatory properties [37]. Phytochemicals present in methanol extracts of Algerian *Hertia cheirifolia* (rich in flavonoids and polyphenols) were tested for their anti-inflammatory effects in an experimental model of croton oil-induced ear edema in albino mice [38]. Topical application of these extracts in the ear of mice inhibited ear swelling caused by croton oil. This treatment also inhibited leucocyte migration and significantly reduced the release of TNF and IL-1β [38].
- Diets supplemented with ellagic acid (Figure 1), a polyphenol found in raspberry seed flour and other fruits or nuts, were effective in reducing high-fat high sucrose-induced metabolic stress by attenuating hepatic endoplasmic reticulum stress, ROS and adipocyte inflammation in mouse [39]. As high fat-high sucrose diet has become part of “western” diet and is the cause of high obesity in western countries, inclusion of ellagic acid either in diet (consummation of fruits and nuts) or as a supplement, potentially might be used to normalize metabolic insult triggered particularly by high sucrose diet. Other berries also deliver beneficial effects against metabolic stress, specifically diabetes mellitus. Thus, consumption of polyphenol-rich white mulberry (*Morus alba*) leaves attenuates diabetic retinopathy in rats [40]. Particularly, rats fed with *M. alba* extracts showed decreased levels of sorbitol, fructose, PKC, and pro-inflammatory cytokines, reduced apoptosis, reduction of Vascular endothelial growth factor (VEGF) expression, and attenuated oxidative stress markers in their retinas [40]. Even more interesting is the fact that *M. alba*-rich diet significantly attenuated hyperglycemia and weight loss. Considering that weight loss is a side effect of pancreatic cancer patients, it might be interesting to test these extracts on rodent models of cachexia. In high fat-fed mice, blood glucose concentration, hepatic metabolism and obesity-related gene expression signature were also improved by the intake of quercetin (Figure 1) and quercetin-containing apple and cherry extracts. In obese high-fat fed mice, phytochemicals from the same fruits also reduced hepatic lipid accumulation, and lowered blood glucose concentration after food deprivation [41]. Finally, extracts of *Acai*-berry inhibited osteoclastogenesis responsible for bone loss and known to be triggered by inflammation. RAW-264.7 macrophages challenged with Receptor activator of nuclear factor kappa-B ligand (RANKL) to induce osteoclastogenesis-like in vitro, showed attenuated osteoclast activity following treatment with *Acai*-berry extracts, as measured by tartrate-resistant acid phosphatase and hydroxyapatite resorption assay [42]. In parallel, *Acai-*berry extracts reduced the secretion of IL-1α, IL-6 and TNF, while increasing the secretion of IL-3, IL-4, IL-13 and interferon gamma (IFN-γ) [42].
- In human umbilical vein endothelial cells (HUVECs), polyphenols-enriched fraction from *Chilean propolis* was shown to inhibit activation of Hif-1α and Erk1/2 as well as expression of VEGFA [43]. Polyphenols are likewise tested for therapeutic prevention of malignant mesothelioma [44]. Malignant mesothelioma is caused by exposure to asbestos that triggers chronic inflammation of mesothelial cells. In addition, asbestos also reduces the ability of the immune system to mount a functional response to transformed mesothelial cells. Current knowledge suggests that polyphenols are able to reduce inflammation and also inhibit cancer cell growth, hence the consideration for using polyphenols for prevention of malignant mesothelioma [44].

## 3. Phytochemicals and microRNAs

MicroRNAs are short, non-coding regulatory RNAs. Their main, although not unique, function, consists in regulating the translation and/or degradation of so-called target messenger RNAs [45,46,47]. Numerous studies have shown microRNAs to regulate fundamental processes, including muscle, cardiac, neural, and lymphocyte development, or the regulation of both the innate and adaptative immune responses [48,49]. Most microRNAs are produced from primary transcripts (pri-miRNAs) that, in the nucleus, are converted into precursor miRNAs (pre-miRNAs) by the RNase III Drosha, associated with DGCR8 to form the small microprocessor complex [50]. Pre-miRNAs subsequently are exported toward the cytoplasm where the RNase III Dicer within the RISC complex cleaves the miRNA hairpin. The guide strand, which corresponds to mature microRNA, is then loaded into the RISC complex [50]. MicroRNAs and their transcriptional regulators usually form auto-regulatory loops aimed at controlling their respective levels [51]. MicroRNAs can directly target several tens to several hundreds of target genes, including factors implicated in signal transduction pathways and transcription factors, which potentially empowers them to indirectly regulate the expression of thousands of genes. This feature makes microRNAs powerful buffering agents, but greatly complicates the study of their actual effects and functions in vivo. As a consequence, microRNA misexpression or malfunction has the potential to negatively affect many gene regulatory networks whose molecular malfunctions are associated with major pathologies such as cancer [47] or auto-immune diseases [49,52,53,54]. In particular, several miRNAs have been shown to play a key role in inflammation and cancer [52,53,54,55]. The most prominent are *miR-155*, *miR-21*, and *miR-125b*. Thus, the expression of *miR-155* is strongly elevated in several human leukemias and lymphomas ([54] and references therein). Transgenic mice with B cells overexpressing *miR-155* develop B-cell leukemia, and a sustained expression of *miR-155* in hematopoietic stem cells causes a myeloproliferative disorder [54]. MicroRNa *miR-21*, aberrantly expressed in a number of cancers, regulates many inflammatory pathways, including TLR signaling [56]. In addition, in chronic lymphocytic leukemia, the down-regulation of *miR-125b*, another microRNA implicated in the regulation of the innate immune response to LPS [55,56], allows B lymphocytes to acquire a metabolism that favors proliferation, reminiscent of the Warburg effect [57]. It is therefore of tremendous interest to study molecules that would be capable to shield the body from exacerbated inflammation by impacting the expression of pro- and anti-inflammatory microRNAs. Interestingly, microRNAs can circulate from cell to cell within exosomes, HDLs or microvesicles, or bound to proteins such as AGO2 [58]. This allows them to deliver effects at a distance, in such a way that they have sometimes been considered as proto-hormones. This means that a drug can potentially change the expression of a microRNA in a particular tissue, with this microRNA exerting its regulatory effects in another tissue after being transported by blood or other body fluids. Finally, it has also been shown that some plant microRNAs may survive for several hours in the gut of mammals due to their 2′-*O*-methylated terminal nucleotide that makes them resistant to oxidation, and subsequently enter the blood, with for example the result of plant *MiR-168a* targeting mammalian *LDLRAP1* transcripts [59]. Nevertheless, it is not very likely that plant microRNAs may act as global regulators of gene expression in animal cells. Namely, Darwinian evolution has forced animal transcripts to co-evolve with animal microRNAs to ensure tight regulation of the genome and efficient maintenance of cell homeostasis.

Microarray microRNA analyses were firstly performed in vitro under different inflammatory conditions to study the effects of phytochemicals on cell microRNAome. Our group first reported that resveratrol treatment of human THP-1 monocytes increases the expression of *miR-663* (Table 1), a microRNA targeting transcripts encoding at least two of the six Activator protein (AP)-1 transcription factors, namely JunB and JunD [60]. AP-1 factors were previously reported to control the expression of *miR-155*-host gene known as *miRHG1*/*Bic* [61]. By impairing the upregulation and activation of AP-1 factors in response to LPS signaling, resveratrol, through *miR-663*, impaired the upregulation of *miR-155* by LPS [60]. *MiR-155* has now been well established to be a critical microRNA of the innate immune response and a pro-inflammatory microRNA [62]. The expression of *miR-155* increases in almost all studied chronic inflammatory pathologies, such as autoimmune disorders, inflammatory bowel syndrome, cardiovascular diseases, or neuro-degenerative disorders such as multiple sclerosis (MS) and amyotrophic lateral sclerosis (ALS) [54,62,63]. We also found that *miR-663* is a target of resveratrol treatment in human SW480 colon cancer cells [19]. In these cells, *miR-663* reduces the levels of *TGF-β1* transcripts and decreases transcriptional activity of SMADs [19] (Table 1). It is critical to note that *miR-663* is only found in Apes but not in rodents [60]. Its homolog, *miR-774*, however, is found in both human and rodents, but whether these two microRNAs are downstream targets of the same signal transduction pathways, or whether they have the same functions in these groups remains elusive. These findings raise cautions when using rodents models, as the results obtained in this group of animals might not get well translated in humans. Finally, resveratrol effects on THP-1 inflammatory response were recently shown to be associated with the up-regulation of *miR-Let7A*, with effects on TNF, IL-6, IL-10, BDNF and ASK1 pathways [64] (Table 1).

Other studies subsequently analyzed the effects of different phytochemicals in other pathological-like conditions. As we have previously reviewed the relations between resveratrol, microRNAs and inflammation [16], in here we will focus on the findings of the last 3–4 years.

As mentioned earlier, *miR-155* is a microRNA whose expression is a target of a myriad of inflammatory signals, with LPS being the most studied one [54,55,65]. Upon its induction, *miR-155* mostly induces a pro-inflammatory response, through the targeting of *Inositol polyphosphate-5-phosphatase* (*INPP5*), also known as *SHIP-1*, or *Suppressor of cytokine signaling* (*SOCS*)*-1* transcripts [66,67]. Therefore, although *miR-155* is considered oncogenic in certain settings, its activity nevertheless is needed for a fully functional immune response. Hence, any phytochemical that increases or decreases *miR-155* expression in a particular cellular setting is of great interest. Following our first studies [19,60], it was shown that pomegranate polyphenols downregulate *miR-155* expression in MCF-10F and MCF12F breast cancer cell lines. This downregulation was simultaneous to the induction of SHIP-1 and impaired activation of PI3K/AKT and NF-κB pathways [68]. Of note, *miR-155* expression also increases in tumors such as leukemias and breast, lung, or gastric cancers [54,62]. Interestingly, *miR-155* was shown to establish a direct mechanistic link between inflammation, and cancer development and progression by enhancing the mutation rate by simultaneously: (i) targeting different genes that suppress mutations; and (ii) decreasing the efficiency of DNA safeguard mechanisms by targeting transcripts encoding WEE1, a kinase that catalyzes the inhibitory tyrosine phosphorylation of Cdc2/Cyclin B, blocking cell-cycle progression at the G_2_/M phase [69]. In addition, in alcoholic liver disease and in inflammatory liver injury, *miR-155* was predominantly associated with the exosome-rich fraction, thus also establishing a new link between inflammation and liver damage [70]. Finally, in mouse, another microRNA, *miR-223*, was found to regulate polarization of bone marrow-derived macrophage in obesity-associated adipose tissue inflammation, by suppressing classic pro-inflammatory pathway and enhancing the alternative anti-inflammatory response, in particular by targeting *Pknox1* [71]. Thus, microRNAs behave as global regulators that provide mechanistic links between inflammation, metabolic diseases and cancer.

Many of the effects of phytochemicals on microRNAome were studied in cardiovascular settings, reflecting the impact of our diet on our cardiovascular health and aiming to discover potential microRNA-based and phytochemical-based preventive treatments. Based on the fact that an unhealthy endothelium induces chronic inflammation, identifying phytochemicals capable to modulate the expression of microRNAs that have anti-inflammatory effects on endothelium is of great interest.

- Polyphenols found in *Chilean propolis* were tested for their anti-angiogenic effects in atherosclerotic plaques from *LDL receptor* knockout mice [35] (Table 1). Propolis attenuated cell migration, capillary-like tube formation and sprouting by inhibiting the activation of Hypoxia-inducible factor 1-alpha (HIF-1α) and ERK1/2 kinases, as well as the expression of Vascular endothelial growth factor (VEGF) [35] (Table 1). Mango extracts that, as mentioned earlier, show anti-inflammatory and anti-oxidant properties suppressed the expression of NF-κB, PI3K (p85β), HIF-1α, p70S6K1, RPS6 and iNOS in LPS-treated CCD-18Co cells [72] (Table 1). Simultaneously, mango extracts had opposing effects on the expression of *miR-126*, with LPS reducing *miR-126* expression and mango extracts increasing it [72]. *MiR-126* was shown to target *PI3K* (*p85β*) transcripts. As most of the transcripts whose expression was changed by mango extracts encode members of the PI3K/AKT/mTOR pathway, it has been suggested that mango extracts impair the activation of this pathway by upregulating *miR-126*. It clearly remains to uncover the specific mechanisms of *miR-126* upregulation in this setting, as *miR-126* is crucial for the normal function of endothelial cells, and its expression is deregulated in several cardiovascular diseases, hence the interest of manipulating the levels of this microRNA [73]. *MiR-126* is likewise the target of other polyphenols such as those derived from cowpea (*Vigna unguiculata*) or from pomegranate, both groups known for their anti-inflammatory properties [74]. Accordingly, the treatment of LPS-challenged colonic myofibroblasts with different polyphenols from cowpea plant impaired the robust induction by LPS of pro-inflammatory factors such as IL-8, TNF, VCAM-1, and NF-κB, while increasing the levels of *miR-126*, that itself targets VCAM [74] (Table 1). Likewise, pomegranate juice given to rats prior to inducing colorectal aberrant crypt foci using azoxymethane reduced azoxymethane effects and lowered the proliferation of mucosa cells by increasing the expression of *miR-126* [75]. Pomegranate-induced *miR-126* reduced the levels of *VCAM-1* and *PI3K p85β* target transcripts as well as the expression of pro-inflammatory cytokines, NF-κB, iNOS and COX-2 [75] (Table 1). Another polyphenol-containing substance similarly acting as an anti-angiogenic factor is propolis. When LDL receptor knockout mice that are prone to atherosclerotic plaques were treated with propolis, atherosclerotic lesions were significantly attenuated and the expression of pro-angiogenic factor VEGF and of Hif-1α was reduced [35] (Table 1). Hif-1α, known to positively control the expression of VEGF at the transcriptional level, was expressed in the necrotic nucleus of atheromas. In addition to the transcriptional control of VEGF, propolis treatment significantly increased the expression of *miR-181a*, *miR-106a* and *miR-20b* [35]. These three microRNAs were found to target and reduce the expression of transcripts encoding both *VEGF* and *Hif-1α*. Thus, anti-angiogenic effects of propolis are at least through these three microRNAs in addition to its effects on protein coding genes.
- *MiR-34* has been an attractive microRNA for its therapeutic potentials against cancer, and *miR-34*-based therapeutics are now on clinical trials [76,77,78]. Honokiol (Figure 1), a polyphenol present in *Magnolia grandiflora*, was found to upregulate the expression of *miR-34a*, which in turn targets and inhibits the Wnt-1-Metastasis-associated protein (MTA)1-β-catenin pathway (Table 1), thus counteracting oncogenic effects of leptin on breast cancer growth and metastatic properties of human breast cancer cell lines MCF7, MDA-MB-231, MDA-MB-468 and T47D [79]. Based on a phospho-kinase screening array, honokiol was found to inhibit the phosphorylation and activation of factors involved in Leptin-signaling. Accordingly, honokiol inhibited the phosphorylation of Stat3, a transcription factor that binds to and represses the expression of *miR-34* [79]. Of note, in breast cancer, Wnt-1-MTA1-β-catenin pathway is activated by Leptin, and is considered a critical therapeutic target. Other polyphenols derived from pomegranate also induced the expression of *miR-34a*, this time in EJ bladder cancer cells, where *miR-34a* was reported to target *c-Myc* and *CD44* transcripts [80] (Table 1).
- Another microRNA involved in inflammation and cancer is *miR-181b* [81]. *MiR-181b* binds to the 3′-UTR of transcripts encoding pro-inflammatory chemokines (C-X-C motif) ligand (CXCL)1 and CXCL2 and simultaneously reduces their expression in metastatic breast cancer cells [82] (Table 1). Curcumin had similar effects on these two transcripts, suggesting that its effects on breast cancer cells are potentially due to its upregulation of *miR-181b* expression [82]. Indeed microRNA microarray analysis of curcumin-treated breast cancer cells showed that this phytochemical upregulates the expression of *miR-181b* and reduces metastatic progression. Curcumin also significantly reduced the growth of flank murine melanoma tumors by substantially upregulating the expression of *miR-205-5p* [83] (Table 1). Of interest to note is also the fact that curcumin altered the expression of microRNAs whose corresponding putative target transcripts are implicated in O-glycan biosynthesis and endoplasmic reticulum protein maturation and/or processing. These results suggest testing curcumin in other types of cancer where *miR-205-5p* and the above-mentioned mechanisms are involved.
- As mentioned earlier, the flavonoid apigenin has anti-viral activity [15]. This activity was attributed to apigenin acting as an inhibitor of microRNA maturation, especially that of *miR-122*, previously shown to positively regulate hepatitis C virus replication [15] (Table 1). In human liver Huh7 cells, apigenin blocked microRNA maturation by preventing phosphorylation and consequently activation of TAR RNA binding protein (TRBP) enzyme, which is involved in microRNA maturation [15]. The impairment of microRNA maturation was associated with the reduction of hepatitis C virus replication. This finding is of great interest for patients chronically infected by hepatitis C virus. In (*miR103*)-overexpressing transgenic mice presenting with glucose intolerance, apigenin improved this pathogenic status likely by decreasing matured *miR103* expression levels through inhibition of TRBP phosphorylation [84] (Table 1). Given the above results, it would certainly be of interest to look for additional compounds with anti-bacterial, anti-viral, or anti-parasite activity that might impact affect the expression of microRNAs capable to inhibit the survival and/or the functions of pathogens.
- With regard to phytochemicals and oxidative stress, it did not come as a surprise that different phytochemicals induce the expression of *miR-210*, the most critical microRNA of oxidative stress response [62,85]. This induction was primarily at the transcription level, as it was found that phytochemicals such as EGCG upregulate the expression of HIF-1α, the transcription factor for *miR-210* [86] (Table 1).
- *MiR-21* is an oncogenic microRNA upregulated during the immune response and highly expressed in nearly all solid and liquid malignancies. Targeted downregulation of *miR-21* therefore is of upmost interest in the field of cancer [45]. Opposite to cancer cells, *miR-21* expression is significantly reduced in ischemic heart, thus restoring its expression in these conditions would be desirable [87]. Therefore, identifying compounds that could either increase or reduce *miR-21* expression is critical. Certainly, phytochemicals should have specific effects based on the cell type, cell condition, cell function, and depending primarily on the cell transcriptome, metabolome, and proteome. Therefore, it is most likely that cell-specific conditions affecting phytochemicals effects would also impact phytochemicals effects on *miR-21* expression. This microRNA has often been associated with the activity of NF-κB [45]. Resveratrol treatment of U251 brain tumor cells induces apoptosis and decreases *miR-21* expression and NF-κB activity [88] (Table 1). On the other hand, *miR-21* was shown to protect the heart from ischemia through its anti-apoptotic activity. In cardiomyocytes, *miR-21* targets and consequently decreases the levels of transcripts encoding Fas ligand, a pro-apoptotic factor [89]. In addition to *miR-21*, another microRNA involved in neuroblastoma is *miR-7-1* [90]. The expression of this microRNA increases in malignant neuroblastoma cell lines following treatment with either EGC or EGCG [90]. Upon its upregulation by EGCG or EGC treatment, *miR-7-1* contributes to the induction of cell apoptosis [90]. The pro-apoptotic activity of EGCG and EGC also arises from their effects on the expression of other microRNAs, including the downregulation of oncogenic microRNAs from the *miR-17-92* cluster family: specifically, *miR-92*, *miR-93* and *miR-106b* [90] (Table 1). This downregulation is probably through modulation of transcription factors that control the expression of this cluster, such as GAM [91].
- A combinatorial-correlative computer based analysis based on a mouse tobacco carcinogen-induced lung tumor model led to the identification of a signature transcripts/microRNAs/pathways associated with EGCG treatments [92], with NF-κB, AKT, and MAP kinase pathways being the major pathways affected by this compound. This study found twelve microRNAs upregulated and nine microRNAs downregulated by EGCG (Table 1).

## 4. The Gut Microbiota and the Bioavailability of Phytochemicals

The human intestinal tract contains up to 100 trillion microbes, most of them being found in the colon. With a microbe density around 10^11^–10^12^ cells/mL, the colon presents with the highest recorded density for any microbial habitat [93]. Some microbes represent permanent residents whose relations with the host are either commensal or mutualistic [93]. In the gut, exposure to the microbiota, food-derived antigens, metabolites, and pathogens represents a formidable challenge that can only be addressed through a highly complex network of regulatory pathways, with the consequence that a great part of immune activity is aimed at controlling the relationship of the body with the microbiota [94]. The core activity of the human gut microbiota is carbohydrate fermentation, which controls carbon utilization and energy production by the colon [95]. The composition of the microbiota in human gut is affected by genetic factors as well as by a number of extrinsic factors such as the mode of infant delivery, neonatal and adult nutrition, antibiotic exposure, stress, age, hygiene and bacterial infections, so that it is believed that each individual develops a unique microbial composition that translates into a unique susceptibility to several diseases [96]. As many pathologies have now been linked with the composition of the gut microbiota, including metabolic and obesity-related diseases, liver diseases, and inflammatory bowels diseases [95,97], it is clear that diet composition may have either a positive or a negative effect on individual health by modifying the composition of gut microbiota, and that any diet with unbalanced composition has the potential to impact the composition of the microbiota and consequently the inflammatory status and the health [96]. Indeed, fecal microbiota transplantation has been developed as a new promising therapeutics for treatment of several gut inflammatory diseases [97]. On the other hand, most polyphenols in plants and food are present as glycosylated derivatives, so that their bioavailability to the host depends on their transformation by enzymes produced by the gut microbiota [96,98]. In the process, microbes produce metabolites that can also impact the composition of gut microbiota, the function and degree of inflammation of the gut, and consequently the health of the gut and of the individual as a whole [96,98,99,100,101,102,103,104]. The ability of plant polyphenols to control the composition of the microbiota and cellular function, and to modulate the inflammatory status, either directly or through their metabolites, is not easy to assess, and most published results have been drawn from experiments consisting in treating cells or bacteria cultures. It is relatively easy to measure the effects of plant polyphenols on cancer (they usually reduce cell proliferation and metastasis while increasing cell death) or on animal models of human pathologies (they produce healthier phenotypes). In contrast, the actual effects of plant polyphenols on inflammation are usually estimated from their capability to reduce cytokine/chemokine production and/or the expression of pro-inflammatory genes. However, while a strong, acute inflammatory response is usually beneficial to the body, at least when it remains contained, chronic, low level inflammation can be very harmful and lead to auto-immune, neuro-degenerative and cardiovascular diseases, or to cancer [49,54,62,63,69]. Thus, it may well be that beneficial anti-inflammatory effects of phytochemicals may result from a long exposure at low doses rather than from short treatment at higher doses. The actual effects of phytochemicals are usually assessed in animal models, with the inconvenience that their microbiota likely is very different from the human microbiota, so that results drawn from studies in animals cannot be easily translated to human. Thus, resveratrol proved beneficial against brain inflammation or brain ischemic injury in different rodent models [105,106,107,108]. Of course, it is better to assess phytochemicals activity in clinical trials in human, with the inconvenience that obvious ethical constraints do not allow to measure many parameters that would be critical to conclude on the presence or absence of beneficial effects, not to mention the cost of these trials and the need for conducting them repeatedly on samples of sufficient size. Nevertheless, resveratrol has shown beneficial effects in particular against metabolic and cardiovascular diseases in human [109,110,111]. Finally, the most convincing pieces of information about potential beneficial effects of plant polyphenols came from epidemiological studies on non-medical subjects: for example, individuals whose diet supplies low but regular doses of resveratrol show an overall improvement of their health as compare with those of control groups [112,113]. Of note, it has been observed that higher doses of plant polyphenols may not always translate into higher activity: for example, enterocytes metabolize resveratrol to glucuronide and sulfate derivatives that are excreted through ABC transporters [114,115,116]. The fact that resveratrol may display high activity while its bioavailability is low has sometime been called “resveratrol paradox” [117]. As a consequence, it is easy neither to identify the “real” actor responsible for resveratrol effects (resveratrol itself, certain derivatives or certain combinations of derivatives) nor to define the optimal dose that could be used for desired effects [118]. For example, flow mediated dilatation, a procedure used to assess endothelial function, do not increase proportionally to resveratrol concentration in non-medicated overweight/obese individuals with mildly elevated blood pressure [119]. Finally, the facts evocated here above for resveratrol are also likely to be valid for other plant polyphenols.

## 5. Closing Remarks

There is a scene at the end of the movie Casablanca, where the chief of police played by the gifted Claude Rains says to the police officers at the scene: “Round up the usual suspects”. At this stage of research dealing with microRNAs, phytochemicals and inflammation, we have done just that. It is now time to study in depth the molecular and cellular effects of specific plant polyphenols on microRNAs expression, either alone or in combination, including studies on their metabolism and on the potential effects of their derivatives. Especially, it will be important to conduct in vivo studies to determine which of these compounds warrants further clinical trial, if any.

One major problem when it comes to the effects of plant polyphenols on microRNAs is that it is very hard to get convincing evidence about mechanisms that allow them to change the pools of endogenous microRNAs. For example, some phytochemicals have been reported to act as chelating agents. Thus, quercetin is a potent iron chelator, and quercetin treatment resulted in reduced duodenal expression of Ferroportin (FPN) [120]. As a consequence, acute exposure of rat duodenal mucosa to quercetin increased apical iron uptake but decreased subsequent basolateral iron efflux into circulation. Evidently, the consumption of quercetin is of interest for patients with excess iron in their body. Besides binding to metals and acting as chelating agents, plant polyphenols could directly bind microRNAs as well. Thus, a recent report established the ability of resveratrol and EGCG to directly bind to *miR-33a* and *miR-122*, and to modulate their levels in liver cells [121]. Furthermore, the binding of resveratrol and EGCG to these two microRNAs was specific, as determined using nuclear magnetic resonance spectroscopy. On the other hand, *miR-33* and *miR-122* expression was repressed in rat hepatocytes treated with grape seed proanthocyanidins [122]. Although the mechanisms of action of grape seed proanthocyanidins on *miR-33* and *miR-122* expression in liver cells were not studied [122], it is highly probable that, like resveratrol and EGCG, grape seed proanthocyanidins may transiently bind to these two microRNAs. Of note, *miR-33* and *miR-122* are highly abundant in liver cells, and thus probably easily accessible by the tested compounds.

Altogether, plant polyphenols display a wide variety of biological activities in relation with the fact that they can either inhibit voltage-gated ion channels, chelate various metals needed for biochemical reactions, react in the oxidized form with nucleophiles present in the side chain of proteins (such as cysteine and lysine), etc. These different properties allow these compounds to affect the behavior of a number of proteins, including enzymes, channels, and others. Due to this multiplicity of properties, plant polyphenols tend to be promiscuously active in independent target-based assays, fluorescence-based assays, cell-based assays and in vivo assays used in present-day massive screenings of bioactive molecules [123,124,125]. Given that crude phytochemicals, most often contain a combination of several plant polyphenols, they behave as polyfactorial agents (multiagents, and multitargets) that challenge the process of fractionation, making it extremely difficult, if not impossible, to identify their “true” target in the cell, i.e., a protein with which they would interact at a high specificity [124,125]. This difficulty is aggravated by the fact that these components often act at very low dose and with an apparent lack of specificity, and, due to their promiscuous effects, do not seem to allow the development of derivatives that would present stronger, more specific activity. This is the reason why some phenolic NPs have often been described either as Pan Assays Interference Compounds (PAINS) or as Invalid Metabolic Panacea (IMPs) [124,125]. This does not, however, mean that these compounds are without activity or devoid of any therapeutic interest. This merely shows that assays used in present automated massive-screenings are not well adapted to the analysis of this kind of molecules, and therefore that such phenolic NPs should rather not be included in the libraries of molecules to be screened in this kind of assays.

It is well possible that, under Darwinian selection pressure, animals, especially vegetarian vertebrates, whose regular diet contains plant polyphenols such as those discussed in this review, have succeeded in using these molecules as environmental cues allowing them to control and maintain cell homeostasis, along with factors such as the duration of day or night, the composition of populations of microorganisms to whom they are exposed, or the physico-chemical characteristics of their environment. Permanent exposure to plant polyphenols could therefore have resulted in genetic changes making these compounds harmless, at least at low dose, and allowing vertebrates to progressively take advantage of different compounds acting at diet doses. This may explain the apparent paradox that increasing the dose of some phenolic NPs does not seem to make their effects greater. As a consequence, it is well possible that plant polyphenols may be extremely important in the prevention of metabolic, cardiovascular or auto-immune pathologies by providing the organism with added buffering capabilities against harmful environments, and at the same time that, due to their lack of specificity, the same products or their derivatives may be of limited interest as curative agents. Of note, the behavior of plant polyphenols is reminiscent of that of microRNAs that have been shown to be active in a small range of concentrations only: for example, *miR-155* has been shown to target *Quaking* transcripts only at low dose on the onset of the inflammatory response [63]. Finally, the fact that microRNAs also display promiscuous activities, due to their potential to target, either directly or indirectly, tens to hundreds of genes implicated in multiple regulatory pathways and to travel from cell to cell, may in part explain why plant polyphenols that are able to modify the composition of microRNA populations have so many, seemingly unspecific and hard-to-assess activities in the body.

Extensive studies conducted during the last decade led to the characterization of the functions of microRNAs that are changed in a number of pathologies, such as metabolic, autoimmune, cardiovascular and neuro-inflammatory diseases or cancers. This work has allowed to identify groups of microRNAs that are potential targets for treatment or prevention of specific pathologies. It would therefore be of primary interest to determine the precise effects of plant polyphenols on these “selected” microRNAs, in order to optimize the use of phytochemicals found in our diet depending on the particular pathological state, the genetic background, etc. Parallel to microRNAs, long non-coding transcripts have gained great attention during the last decade. Clear evidence suggests that these long non-coding transcripts, like microRNAs, have a number of effects on cell homeostasis, especially, although not exclusively, through epigenetic modifications. Expectedly, plant polyphenols might harbor the potential to change the expression of some non-coding transcripts, which would be of tremendous importance for their use in therapeutics.

Medications derived from some plant polyphenols, normally found in our diet but possibly not at adequate concentration, have now become a big, profitable pharmacy business. In California, a yearly supply of resveratrol—that people are taking as a cancer preventive and anti-inflammatory supplement—amounts to $40,000. However, studies in human are still limited. Although the paradox of French diet can be used as an argument for positive effects of resveratrol on our health, the genetic background also should be taken into consideration. Hence, the need for developing studies directed on the effects of plant polyphenols in specific genetic background and at different stages of the progression of major pathologies to elucidate the optimal conditions for the use of these compounds. As described here above, resveratrol exerts strong anti-inflammatory effects in particular, although not exclusively, by reducing the levels of *miR-155* by downregulating the expression of AP-1 factors [60], or, in the light of recent results [121], also possibly by directly binding to *miR-155* and reducing its activity (which remains to be shown). Nevertheless, we should not forget that *miR-155* is critical for mounting a functional immune response toward infecting agents or tumor emerging cells. Therefore, the dose and duration of any treatment with a given plant polyphenol will have to be optimized to favor positive effects against deleterious ones. Finally, a few recent reports also suggested that phytochemicals might have effects on fetal epigenome [126]. While some phytochemicals might be of benefit to the fetus, their excessive use during pregnancy potentially could also have adverse effects at a later stage of life and, again, caution must be taken.

In conclusion, it is probable that it is not the consumption of plant polyphenols per se, but their consumption in the presence of an adequate, healthy gut microbiota diversity that is key to the beneficial effects of these products to the health [98]. These effects on the gut microbiota may well provide a partial explanation to the conundrum of low oral bioavailability yet high bioactivity of resveratrol or other traditional herbs [127]. Furthermore, intricate interactions between plant polyphenols and the gut microbiota might at least in part explain why these compounds can deliver effects that are not proportional to the ingested dose, as in the case of the “resveratrol paradox” [117]. This characteristic may possibly result from the activity of enzymes produced by inducible operons encoded by the genome of some microbes present in the gut. Namely, increasing phenolic NP oral dose would expectedly result in an ever greater activation of the transcription of genes present in these operons, and therefore an ever greater production of enzymes implicated in the metabolism of these compounds. Finally, the fact that low concentrations of plant polyphenols can still deliver a high activity may at least in part result from the fact that these polyphenols have the ability to modify the composition of microRNA populations within cells, and especially enterocytes. Consequently, even small changes in the levels of certain critical microRNAs may translate, directly and indirectly, into modifications of the expression of tens to hundreds of genes belonging to multiple pathways not only in gut cells, but also in cells from other organs as a result of microRNAs travelling from cell to cell. Finally, the fact that the effects of plant polyphenols are ultimately beneficial most probably results from evolutionary pressures that have ensured coordinated changes in the sequence of microRNAs and of their target transcripts as well as in cell machinery and metabolism. Ultimately, this co-evolution process has led to an astonishing situation where low doses of plant polyphenols seemingly happen to produce a great number of beneficial effects.

## Figures and Tables

**Table 1:** MicroRNAs implicated in inflammatory response whose expression was modified by different phenolic natural products, and their main target transcripts and pathways.

Molecules	Effects on microRNAs	Cells/Animals	Main Target Transcripts or Pathways	References
Resveratrol	*miR-663* up	THP-1 cells	*JunD* and *JunD*; Impairs *miR-155* up-regulation	[60]
Resveratrol	*miR-663* up	SW480 cells	*TGF-β1* transcripts and SMAD2/SMAD3/SMAD4 pathway	[19]
Resveratrol	*miR-Let7A* up	THP-1 cells	TNF, IL-6, IL-10, BDNF and ASK1 pathways	[64]
Resveratrol	*miR-21* down	U251 brain tumor cells	NF-κB pathway	[88]
Pomegranate polyphenols	*miR-155* down	MCF-10F and MCF-12F cells	PI3K/AKT and NF-κB pathways	[68]
*Chilean propolis* polyphenols	*miR-20b*, *miR-106a*, and *miR-181a* up	Atherosclerotic plaques of LDL receptor knockout mice	HIF-1α, ERK1/2 and VEGF pathways	[35]
Mango extracts	*miR-126* up	CCD-18Co cells	*PI3K*(*p85β*) transcripts and PI3K/AKP/mTOR pathway	[72]
Cowpea (*Vigna unguiculata*) polyphenols	*miR-126* up	CCD-18Co cells	*VCAM-1* transcripts; TNF, IL8 and NF-κB pathways	[74]
Pomegranate juice polyphenols	*miR-126* up	Colon mucosa of rats injected with azoxymethane (AOM) subcutaneously	*PI3K*(*p85β*) and *VCAM-1* transcripts; iNOS, COX-2 and NF-κB pathways	[75]
Honokiol	*miR-34a* up	Human breast cancer cell lines	*Wnt1* transcripts; Wnt1-MTA1-β-catenin pathway	[79]
Pomegranate rind polyphenols	*miR-34a* up	EJ bladder cancer cells	*c-Myc and CD44* transcripts	[80]
Curcumin	*miR-181b* up	MDA-MB-231 human breast cancer cells	*CXCL1* and *CXCL2* transcripts	[82]
Curcumin	*miR-205-5p* and other microRNAs up	Murine melanoma	Proliferation and metastatic pathways; O-glycan biosynthesis; Endoplasmic reticulum protein maturation and/or processing	[83]
Apigenin	*miR-122* down	Human Huh7 cells	*miR-122* positively regulates hepatitis C replication in enhancing viral translation	[15]
Apigenin	*miR-103* down	(*miR103*)-overexpressing transgenic mic presenting with glucose intolerance	TRBP phosphorylation; Improvement of pathogenic status	[84]
EGCG	*miR-210* up	Mouse lung adenocarcinoma cell line CL13	Pathways implicated in cell-proliferation and anchorage-independent growth	[86]
EGCG or EGC	*miR-7-1* up, and *miR-92*, *miR-93*, and *miR-106b* down	SH-SY5Y and SK-N-DZ neuroblastoma cell lines	Apoptosis pathway	[90]
EGCG	12 microRNAs up, and 9 microRNAs down	A/J mice lung adenoma	AKT, MAP kinases and NF-κB and cell cycle regulation pathways	[92]
